# Supplementary material for: Effectiveness of mental health interventions for older adults in South Asia: A scoping review
Source: PLoS One. 2023 Jul 7;18(7):e0287883. doi: 10.1371/journal.pone.0287883 (PMC10328249; doi:10.1371/journal.pone.0287883)
Supplement: S1 File — (DOCX) [file pone.0287883.s002.docx]

**Detail search strategy in Medline:**

| Name of database | Keywords were searched in Medical Subject Headings (MeSH) and title and abstract |
| --- | --- |
| Medline | 1. "older adult*" |
|  | 1. “elder* people” |
|  | 1. “elderly” |
|  | 1. “aging” |
|  | 1. “old* people” |
|  | 1. "geriatric*" |
|  | 1. "gerontolog*" |
|  | 1. 1 OR 2 OR 3 OR 4 OR 5 OR 6 OR 7 |
|  | 1. “mental health” |
|  | 1. “mental wellbeing” |
|  | 1. “mental well-being” |
|  | 1. “mental disorder*” |
|  | 1. “mental illness” |
|  | 1. “psychiatr*” |
|  | 1. “psychological health” |
|  | 1. “psychological distress” |
|  | 1. “psychological impact” |
|  | 1. “psychological outcomes” |
|  | 1. “psychological consequence*” |
|  | 1. “psychological comorbid*” |
|  | 1. “psychosocial problem*” |
|  | 1. “behavioral problem*” |
|  | 1. “behavioral disorder*” |
|  | 1. “cognitive disorder*” |
|  | 1. “cognitive impairment*” |
|  | 1. “emotional distress” |
|  | 1. “depression” |
|  | 1. “depressive disorder*” |
|  | 1. “anxiety” |
|  | 1. “PTSD” |
|  | 1. “PTSS” |
|  | 1. “posttraumatic*” |
|  | 1. “post-traumatic*” |
|  | 1. “addiction” |
|  | 1. “substance use disorders” |
|  | 1. “mood disorder*” |
|  | 1. “affective disorder*” |
|  | 1. “DSM*” |
|  | 1. “psychosis” |
|  | 1. “psychotic” |
|  | 1. “oppositional defiant disorder” |
|  | 1. “hyperactiv*” |
|  | 1. “conduct disorder” |
|  | 1. “obsess*” |
|  | 1. “phobi*” |
|  | 1. “schizophren*” |
|  | 1. “bipolar disorder” |
|  | 1. “anorexia” |
|  | 1. “bulimi*” |
|  | 1. “challenging behav*” |
|  | 1. 9 OR 10 OR 11 OR 12 OR 13 OR 14 OR 15 OR 16 OR 17 OR 18 OR 19 OR 20 OR 21 OR 22 OR 23 OR 24 OR 25 OR 26 OR 27 OR 28 OR 29 OR 30 OR 31 OR 32 OR 33 OR 34 OR 35 OR 36 OR 37 OR 38 OR 39 OR 40 OR 41 OR 42 OR 43 OR 44 OR 45 OR 46 OR 47 OR 48 OR 49 OR 50 |
|  | 1. “intervention*” |
|  | 1. “program*” |
|  | 1. “polic*” |
|  | 1. “therap*” |
|  | 1. “counsel*” |
|  | 1. “manag*” |
|  | 1. "cognitive behavio*" |
|  | 1. 52 OR 53 OR 54 OR 55 OR 56 OR 57 OR 58 |
|  | 1. “Afghan∗” |
|  | 1. “Bangladesh∗” |
|  | 1. “Bhutan∗” |
|  | 1. “India∗” |
|  | 1. “Maldiv∗” |
|  | 1. “Nepal∗” |
|  | 1. “Pakistan∗” |
|  | 1. “Sri Lanka∗” |
|  | 1. “South Asia∗” |
|  | 1. 60 OR 61 OR 62 OR 63 OR 64 OR 65 OR 66 OR 67 OR 68 |
|  | 1. 8 AND 51 AND 59 AND 69 |

**Detail search strategy in remaining four databases:**

| Name of database | Keywords were searched for retrieving literature |
| --- | --- |

| Name of database | Keywords were searched for retrieving literature |
| --- | --- |
| APA PsycInfo, Academic Search Ultimate, CINAHL, Health Policy reference Center and Web of Sciences | 1. "older adult*" |
|  | 1. “elder* people” |
|  | 1. “elderly” |
|  | 1. “aging” |
|  | 1. “old* people” |
|  | 1. "geriatric*" |
|  | 1. "gerontolog*" |
|  | 1. 1 OR 2 OR 3 OR 4 OR 5 OR 6 OR 7 |
|  | 1. “mental health” |
|  | 1. “mental wellbeing” |
|  | 1. “mental well-being” |
|  | 1. “mental disorder*” |
|  | 1. “mental illness” |
|  | 1. “psychiatr*” |
|  | 1. “psychological health” |
|  | 1. “psychological distress” |
|  | 1. “psychological impact” |
|  | 1. “psychological outcomes” |
|  | 1. “psychological consequence*” |
|  | 1. “psychological comorbid*” |
|  | 1. “psychosocial problem*” |
|  | 1. “behavioral problem*” |
|  | 1. “behavioral disorder*” |
|  | 1. “cognitive disorder*” |
|  | 1. “cognitive impairment*” |
|  | 1. “emotional distress” |
|  | 1. “depression” |
|  | 1. “depressive disorder*” |
|  | 1. “anxiety” |
|  | 1. “PTSD” |
|  | 1. “PTSS” |
|  | 1. “posttraumatic*” |
|  | 1. “post-traumatic*” |
|  | 1. “addiction” |
|  | 1. “substance use disorders” |
|  | 1. “mood disorder*” |
|  | 1. “affective disorder*” |
|  | 1. “DSM*” |
|  | 1. “psychosis” |
|  | 1. “psychotic” |
|  | 1. “oppositional defiant disorder” |
|  | 1. “hyperactiv*” |
|  | 1. “conduct disorder” |
|  | 1. “obsess*” |
|  | 1. “phobi*” |
|  | 1. “schizophren*” |
|  | 1. “bipolar disorder” |
|  | 1. “anorexia” |
|  | 1. “bulimi*” |
|  | 1. “challenging behav*” |
|  | 1. 9 OR 10 OR 11 OR 12 OR 13 OR 14 OR 15 OR 16 OR 17 OR 18 OR 19 OR 20 OR 21 OR 22 OR 23 OR 24 OR 25 OR 26 OR 27 OR 28 OR 29 OR 30 OR 31 OR 32 OR 33 OR 34 OR 35 OR 36 OR 37 OR 38 OR 39 OR 40 OR 41 OR 42 OR 43 OR 44 OR 45 OR 46 OR 47 OR 48 OR 49 OR 50 |
|  | 1. “intervention*” |
|  | 1. “program*” |
|  | 1. “polic*” |
|  | 1. “therap*” |
|  | 1. “counsel*” |
|  | 1. “manag*” |
|  | 1. "cognitive behavio*" |
|  | 1. 52 OR 53 OR 54 OR 55 OR 56 OR 57 OR 58 |
|  | 1. “Afghan∗” |
|  | 1. “Bangladesh∗” |
|  | 1. “Bhutan∗” |
|  | 1. “India∗” |
|  | 1. “Maldiv∗” |
|  | 1. “Nepal∗” |
|  | 1. “Pakistan∗” |
|  | 1. “Sri Lanka∗” |
|  | 1. “South Asia∗” |
|  | 1. 60 OR 61 OR 62 OR 63 OR 64 OR 65 OR 66 OR 67 OR 68 |
|  | 1. 8 AND 51 AND 59 AND 69 |
