# Supplementary material for: Effectiveness of mental health interventions for older adults in South Asia: A scoping review
Source: PLoS One. 2023 Jul 7;18(7):e0287883. doi: 10.1371/journal.pone.0287883 (PMC10328249; doi:10.1371/journal.pone.0287883)
Supplement: Attachment — Submitted filename: MH Interventions - PLOS ONE.pdf [file pone.0287883.s005.pdf]

To the editor:

As a result of the review, my opinion is that:

- The article appears to be relevant to the journal. The study brings new contributions to Mental Health interventions
- This manuscript that the authors have done is interesting and opens doors for future research.
- It’s a relevant and current topic, general fulfilling the steps of a scoping review
- However, there are some issues that need to be addressed to improve the quality of the manuscript that are indicated in the comments to the author.
- I believe it should be published after minor changes.

To the autors:

Thank you for giving me the oportunity to review this manuscript entitled “Mental Health Interventions for older adults in South Asia: a scoping review”.

In my opinion, it’s an interesting manuscript and opens doors for future research. It’s a relevant and current topic, general fulfilling the steps of a scoping review

As for the manuscript, there are some comments or suggestions for alteration:

1 - Title:

- There seem to be two titles:

(i) "Mental Health Interventions for Older People in South Asia: A Scoping Review";

(ii) "Effectiveness of mental health interventions for older adults in South Asia: A scoping review".

The second title is more complete and better reflects the content of the article.

- The keywords reflect the content; however, I suggest you include "effectiveness" or "outcomes" or "impact" or "health gains"

2 - Introduction:

- It's clear, short and simple.
- The introduction reveals an adequate understanding of the relevant literature in the field and cites an appropriate range of literature sources.

3 - Methodology:

The methods employed are adequate.

However:

- A supplementary file with the PRISMA-ScR should be submitted

- Quality/risk of bias assessment of included studies is missing

4 - Results:

The results are clearly presented and properly analyzed.

However, there is the inclusion of a study with an intervention that can be considered a pharmacological intervention; however, there is an eligibility criterion that excludes studies with pharmacological interventionsI suggest that the results and conclusions clearly relate to the review question, namely the conceptualization and measurement of well-being.

As this is a scope review, I suggest that the gap found and the measures to be overcome be presented more clearly and explicitly.
