# Supplementary material for: Effectiveness of mental health interventions for older adults in South Asia: A scoping review
Source: PLoS One. 2023 Jul 7;18(7):e0287883. doi: 10.1371/journal.pone.0287883 (PMC10328249; doi:10.1371/journal.pone.0287883)
Supplement: Attachment — Submitted filename: PLOS ONE Effectiveness of mental health interventions for older adults in South Asia - A scoping review.pdf [file pone.0287883.s006.pdf]

PLOS ONE

Effectiveness of mental health interventions for older adults in South Asia: A scoping review

April 2023

This is an interesting paper providing a scoping review of mental health interventions in South Asia, aiming to synthesize evidence regarding their impact on geriatric mental disorders and highlight research gaps. The study seems to be the result of systematic and careful effort. I therefore think that it deserves publication in the journal, on the condition of important revisions made. The revisions I suggest below concern several clarifications in the text, as well as some considerations mainly on Methodology and Results.

I would like to start with a disclaimer: I am not an expert in the field of mental health interventions for older adults and therefore I cannot comment on the relevance of the literature drawn upon and the quality of contribution regarding the region of South Asia specifically.

Below, I outline the areas that I think are in need of revision:

- According to Sieber (2007), medical treatment of the elderly (geriatrics) starts from the age of 65 years old (https://pubmed.ncbi.nlm.nih.gov/17934704/). Could you please explain why relevant research in South Asia includes adults from the age of 50 years old? Do the terms "older adults" and "elderly" refer to the same age range?
- Pg. 4: "*Mental health deeply encompasses all aspects of human lives, including social, cultural, religious, spiritual, historical, and holistic, which may incur stressors of varying extents.*"
  What exactly do you mean by the term "holistic"? It seems to be a pleonasm, unless it has a different meaning from "*all aspects of human lives*".
- Pg. 4: "*These stressors can result in social alienation, loneliness, and psychosocial distress among older adults that may eventually lead to various mental and behavioral disorders, thereby impacting the overall geriatric quality of life.*"
  Could you please add a reference?
- Pg. 5: "*In addition, socioeconomic and cultural dimensions of growing geriatric mental health problems warrant different psychosocial and community-level interventions that are contextually appropriate to develop an older-friendly environment and social support network.*"
  Could you please provide data supporting this statement, either from South Asia or from other regions or continents?
- Pg. 5: "*To the best of our knowledge, there is a lack of evidence-based reviews exploring mental health interventions among South Asian older adults.*"
  Are there relevant reviews for other regions or continents? If so, could you please provide their findings, in order to compare them with yours in Discussion and enrich your suggestions?

- Pg. 5: "*For scholarly data, we systematically searched Medline, American Psychological Association (APA) PsycInfo, Academic Search Ultimate, Cumulative Index to Nursing and Allied Health Literature (CINAHL), Health Policy Reference Center, and the Web of Science databases from the date of inception of each database up to August 5, 2022, using the specific set of keywords applied with Boolean operators (i.e., "OR," "AND")*".
  Could you please specify the "*date of inception*"?
- Pg. 5-6: "*The search queries were used across titles, abstracts, subject-specific keywords, and topics fields in respective databases*."
  Could you please specify if you set any filter regarding the date of publication etc.?
- Please, correct the page numbering.
- Table 1: Is the search strategy and the keywords presented exactly the same for all the databases that were used? If not, I suggest that you provide an abstract description of the terms that you used in the main text of Methodology and present the database you refer to as an example in Table 1.
- Table 1: Why did you choose "*cognitive behavio**" as a keyword? For example, is there evidence that CBT is more effective or usual than other psychotherapeutic approaches in this field? If so, please add the respective information in the Introduction. Similarly, in the Introduction you could add information on every disorder you chose to include in your keywords (e.g., anorexia, schizophrenia), always regarding older adults.
- Eligibility criteria: Please, specify if you included both quantitative and qualitative research, as well as case studies/practice papers, or if you set any limitations regarding method.
- Eligibility criteria: Please, specify if there were any limitations regarding sex, gender, ethnicity, physical/mental state, or other demographics that you may consider important.
- Eligibility criteria: "*Studies that presented one or more psychosocial, behavioral, or non-pharmacological intervention/s for geriatric mental health problems were included in this review.*"
  Could you please provide the definitions of "*psychosocial therapy/ intervention*" and "*behavioral intervention*" in the Introduction and explain how these apply to older adults (e.g., their main focus or components)?
- Data extraction: "*We used a pre-designed data extraction form that included items on study characteristics…*"
  Was the form designed by the authors? If so, could you please clarify why you did not prefer a standardized form like PICO or SPIDER?
- Characteristics of the included studies: Please provide information regarding the sex/gender of the participants, as well as their physical/mental/emotional state if mentioned in the studies.

- Characteristics of the included studies: Please, specify if there were any Randomised Controlled Trials.
- Table 2: Please, note the statistical significance of the findings, where appropriate. ("*" for p<.05, "**" for p<.01, "***" for p<.001).
- Table 2: Please, explain the abbreviations below the Table in the form of an annotation.
- Table 2: A briefer description of the data would be preferable. Perhaps, you could add further details about the studies in the main text. Also, arranging the studies based on their method and/or therapeutic approach would be helpful for the reader.
- Results: It would be better if you presented the interventions along with their outcomes in separate subsections based on their approach.
- Results: Although not necessary for a scoping review, it would be interesting if you assessed the quality of the studies, based on established criteria for Empirically Supported Theapies (e.g., Chambless et al., 1998), and present their limitations. In that way, it would be easier to identify methodological gaps and make fruitful suggestions for future studies.
- Discussion: "*Scoping reviews are useful for mapping information from existing literature on a certain topic to a broader extent, including research findings and gaps.*"
  This sentence is not necessary.
- Discussion: "*Yoga, Tai chi, and other meditative movements have historically been practiced in South Asia, predominantly assigned as an intervention to explore their effectiveness on geriatric mental disorders.*"
  Could you please provide a reference?
- Discussion: "*Exploring and summarizing evidence…which were likely to increase the risk of bias.*"
  Again, it is suggested that methodology is evaluated more systematically through established criteria for Empirically Supported Therapies (see for example Chambless et al., 1998).
- Discussion: "*These interventions mainly focused on geriatric mental health outcomes, where associated physical, psychological, socioeconomic, and cultural factors remained largely unspecified*."
  Is there relevant literature - even from other regions of the world - that highlights the importance of these factors in geriatric mental health care?
- Discussion: "*However, it has been evident that mental health care utilization among older people is low (59–61); hence, assessing and improving geriatric mental health, especially in low- and middle income countries, including South Asia, becomes critical (62).*"
  Is there any evidence or assumption on why mental health care utilization among older people is low? How exactly can assessment and improvement of geriatric mental health solve this issue? Any ideas from the studies included in your research?

- Discussion: “*The cumulative impact and early-life stressors caused by post-colonial sociocultural reforms in India and Pakistan… and living patterns which compelled older adults to live independently.*”

  Could you please provide references?
- Discussion: “*therefore, we encourage future researchers to adopt more robust methodological approaches in designing intervention studies on geriatric mental health.*”

  How do you interpret the methodological limitations? Do you have any suggestion on specific ways of resolving them? Could you compare the different therapeutic approaches in terms of method, outcomes and effectiveness?
- Proof reading by a native English speaker is recommended.
