# Supplementary material for: Effectiveness of mental health interventions for older adults in South Asia: A scoping review
Source: PLoS One. 2023 Jul 7;18(7):e0287883. doi: 10.1371/journal.pone.0287883 (PMC10328249; doi:10.1371/journal.pone.0287883)
Supplement: Attachment — Submitted filename: PLOS_PONE-D-22-29794.docx [file pone.0287883.s003.docx]

The authors aimed to perform a scoping review to explore the “Mental health interventions for older adults in South Asia: A scoping review” which is relevant and need of the hour. The manuscript looks fine. However, following issues needs to be addressed.

**Title:**

1. Title looks incomplete: It can be “Impact of MH on older adult……..

**Abstract**

2. The abstract can be written in a structured format (it is fine if it depends on authors guideline)
3. Line No: 5; This sentence “for observational/experimental studies evaluating the impact on geriatric mental health in eight countries in the South Asia region” looks incomplete
4. Write a proper conclusion by authors from their observations before the conclusion

**Introduction**

5. Introduction looks fine, written well

**Methodology**

6. A “Preferred Reporting Items for Systematic reviews and Meta-Analyses extension for Scoping Reviews (PRISMA-ScR) checklist should be submitted as a supplementary file
7. Disclose the information on registration of protocol for this review
8. Keep the reference for the Eligibility criteria; point b) for Old age threshold
9. Methodological Quality/risk of bias assessment of included studies is missing

**Results**

10. The inclusion criteria specify the studies to be “non-pharmacological” and authors included the Ayurvedic/herbal medicine (or preparations), which can be considered as pharmacological treatment. Please justify or revise accordingly
11. To say this is a scoping review, authors should add a section describing what is the gap and measures to overcome in the results section. Or else this looks like a systematic review

**Overall**

12. The English Language can be improved further
