# Supplementary material for: Effectiveness of mental health interventions for older adults in South Asia: A scoping review
Source: PLoS One. 2023 Jul 7;18(7):e0287883. doi: 10.1371/journal.pone.0287883 (PMC10328249; doi:10.1371/journal.pone.0287883)
Supplement: Attachment — Submitted filename: Response to Reviewers.docx [file pone.0287883.s004.docx]

**Responses to the Reviewers**

1. Please ensure that your manuscript meets PLOS ONE's style requirements, including those for file naming. The PLOS ONE style templates can be found at <https://journals.plos.org/plosone/s/file?id=wjVg/PLOSOne_formatting_sample_main_body.pdf> and
   <https://journals.plos.org/plosone/s/file?id=ba62/PLOSOne_formatting_sample_title_authors_affiliations.pdf>

Thank you for your guidance. We have revised and formatted our manuscript using PLOS ONE’s style requirements.

2. "PLOS requires an ORCID iD for the corresponding author in Editorial Manager on papers submitted after December 6th, 2016. Please ensure that you have an ORCID iD and that it is validated in Editorial Manager. To do this, go to ‘Update my Information’ (in the upper left-hand corner of the main menu), and click on the Fetch/Validate link next to the ORCID field. This will take you to the ORCID site and allow you to create a new iD or authenticate a pre-existing iD in Editorial Manager. Please see the following video for instructions on linking an ORCID iD to your Editorial Manager account: <https://www.youtube.com/watch?v=_xcclfuvtxQ>"

Thank you for bringing this to our notice. We have linked an ORCID ID of the corresponding author to the Editorial Manager.

3. We note that you have referenced (ie. Bewick et al. [5]) which has currently not yet been accepted for publication. Please remove this from your References and amend this to state in the body of your manuscript: (ie “Bewick et al. [Unpublished]”) as detailed online in our guide for authors
   <http://journals.plos.org/plosone/s/submission-guidelines#loc-reference-style>

Thank you for this comment. We have reviewed our manuscript and found no unpublished article; however, we found one article where reference needed to be updated. Therefore, we have included all information related to that article’s publication in the reference section [reference 18].

4. "In your Data Availability statement, you have not specified where the minimal data set underlying the results described in your manuscript can be found. PLOS defines a study's minimal data set as the underlying data used to reach the conclusions drawn in the manuscript and any additional data required to replicate the reported study findings in their entirety. All PLOS journals require that the minimal data set be made fully available. For more information about our data policy, please see <http://journals.plos.org/plosone/s/data-availability>.

Thank you for this comment. All data associated with this manuscript has been presented as a part of this review.

**Title:**

1. Title looks incomplete: It can be “Impact of MH on older adult……..

Thank you for bringing this to our notice. We have revised our manuscript's title and rephrased some areas throughout the manuscript to keep in line with the study title.

**Abstract**

2. The abstract can be written in a structured format (it is fine if it depends on authors guideline)

Thanks to the Editor for this important feedback. We have reviewed some scoping reviews published in PLOS ONE and revised the abstract of this manuscript accordingly (track-changed part).

3. Line No: 5; This sentence “for observational/experimental studies evaluating the impact on geriatric mental health in eight countries in the South Asia region” looks incomplete.

Thank you for this comment. We have rephased this sentence in the revised version.

4. Write a proper conclusion by authors from their observations before the conclusion.

Thank you for bringing this to our notice. We have added a conclusive statement from our study observation in the conclusion part of the revised abstract.

**Introduction**

5. Introduction looks fine, written well.

Thank you so much. I appreciate this feedback.

**Methodology**

6. A “Preferred Reporting Items for Systematic reviews and Meta-Analyses extension for Scoping Reviews (PRISMA-ScR) checklist should be submitted as a supplementary file.

Thanks to the Editor. I uploaded a copy of PRISMA-ScR checklist in the supplementary file.

7. Disclose the information on registration of protocol for this review.

Thank you for this feedback. We disclosed the information regarding protocol registration of this review in the method under the “Data sources and guidelines” sub-section of the methods.

8. Keep the reference for the Eligibility criteria; point b) for Old age threshold

Thank you for this comment. We have rephrased the sentence to bring more clarity and kept the references with it.

9. Methodological Quality/risk of bias assessment of included studies is missing.

Thanks to the Editor for this comment. In this review, we did not conduct any methodological quality/ risk of bias assessment of included studies. According to JBI manual, Scoping review can be used to map the available evidence, identify/ clarify key concepts in a given field (1,2). It is useful to undertake as a preliminary exercise or for examining evidence when there is a clear knowledge gap exists (1–3). Since scoping review intends to map existing evidence in a broader extent rather than synthesizing clinically meaningful information, therefore critical appraisal or risk of bias assessment is not recommended in the existing scoping review guidelines (3,4) and we mentioned it in our study limitation (in the last paragraph of the discussion section).

**Results**

10. The inclusion criteria specify the studies to be “non-pharmacological” and authors included the Ayurvedic/herbal medicine (or preparations), which can be considered as pharmacological treatment. Please justify or revise accordingly.

Thank you for bringing this to our notice. We included any psychosocial, behavioral, or non-pharmacological interventions in this review. We considered it eligible if any psychosocial intervention was applied in combination with pharmacological treatment or assigned as a comparator to see the effectiveness of psychosocial/ behavioral therapies. That’s why we included those interventions which offered yoga or other meditative intervention in combination with ayurvedic medicine. We have revised the eligibility criteria in the method section to reflect this approach with more clarity.

11. To say this is a scoping review, authors should add a section describing what is the gap and measures to overcome in the results section. Or else this looks like a systematic review.

Thank you for bringing it to our notice. According to guidelines, a scoping review map and present existing evidence in the result section and discussion part allows for a summary of the main results, general interpretation, potential limitations, and measures. Therefore, we presented existing evidence in the result section and summarized all evidence, interpretation, limitation, and potential implications/ measures in the discussion section. To address this feedback, we have added a section to briefly discuss the key research gap identified by this review in the result section (last paragraph of the results section).

**Overall**

12. The English Language can be improved further.

Thank you for this comment. The manuscript has been reviewed by a native English speaker and revised accordingly.

**Reference list:**

1.	11.1.1 Why a scoping review? - JBI Manual for Evidence Synthesis - JBI Global Wiki [Internet]. [cited 2023 Jan 24]. Available from: https://jbi-global-wiki.refined.site/space/MANUAL/4687794/11.1.1+Why+a+scoping+review%3F

2.	Arksey H, O’Malley L. Scoping studies: towards a methodological framework. Int J Soc Res Methodol [Internet]. 2005 Feb 1 [cited 2023 Jan 24];8(1):19–32. Available from: https://doi.org/10.1080/1364557032000119616

3.	Peters MDJ, Godfrey CM, Khalil H, McInerney P, Parker D, Soares CB. Guidance for conducting systematic scoping reviews. JBI Evid Implement [Internet]. 2015 Sep [cited 2023 Jan 24];13(3):141. Available from: https://journals.lww.com/ijebh/FullText/2015/09000/Guidance_for_conducting_systematic_scoping_reviews.5.aspx

4.	Peters MDJ, Marnie C, Tricco AC, Pollock D, Munn Z, Alexander L, et al. Updated methodological guidance for the conduct of scoping reviews. JBI Evid Synth. 2020 Oct;18(10):2119–26.
