# Supplementary material for: Effectiveness of mental health interventions for older adults in South Asia: A scoping review
Source: PLoS One. 2023 Jul 7;18(7):e0287883. doi: 10.1371/journal.pone.0287883 (PMC10328249; doi:10.1371/journal.pone.0287883)
Supplement: Attachment — Submitted filename: Response to Reviewers-14th_June.docx [file pone.0287883.s008.docx]

**Response to reviewer’s comments**

Reviewer #3: I think the authors have addressed my comments in an acceptable way, and, in my opinion, improving the paper. There are a few questions left that the authors should answer:

1. The total number of articles assessed for inclusion in abstract and results section is not similar.

Thanks to the reviewer for this comment. We revisited our manuscript and found consistency in the abstract and result section. In the abstract, we reported, "From a total of 3432 potential articles retrieved, 19 were included in this review following pre-determined eligibility criteria" (Refer to 1st line of the results in the abstract). In the results section, we reported similarly – "We initially found 26 potential citations for full-text evaluation and finally included 19 articles in this scoping review (31–49)" (Refer to the 5-6 lines in 1st paragraph in Results section).

2. "The rapid technological advancement in medical sciences since the 19th century has created different avenues in preventive, promotive, and curative healthcare." Do you mean 19th or 20th century?

Thanks to the reviewer for this important comment. We completely agree with you and revised our manuscript accordingly. Technological advancement in medical sciences started evolving in the 20th century. It gradually expanded in different fields of medical sciences, offering direct services to people with mental disorders (First line in 4th paragraph of Discussion section). Thanks again for bringing this to our notice.

3. Typo: about the effectiveness on metal health outcomes

Thanks to the reviewer. We appreciate this comment. We have addressed the typo, which is now mentioned correctly in the revised manuscript (Refer to the last sentence of the 2nd paragraph in the Discussion section).
